# The U-shaped association between the metabolic score for insulin resistance and incident gallstone disease: a population-based cohort study

**DOI:** 10.3389/fendo.2026.1804048

**Published:** 2026-03-27

**Authors:** Yunxiang Ming, Yangxuan He, Jiayi Deng, Fei Xu, Hong Chen, Yilin Zhu, Jingshan Jiang, Yang Liu, Song Leng

**Affiliations:** 1 Health Management Center, The Second Hospital of Dalian Medical University, Dalian, China; 2 Department of Gastroenterology, The Second Hospital of Dalian Medical University, Dalian, China

**Keywords:** cohort study, gallstone disease, health examination population, METS-IR, U-shaped association

## Abstract

**Background:**

The association between the Metabolic Score for Insulin Resistance (METS-IR) index and incident gallstone disease remains unclear. This study investigated this association using cohort data.

**Methods:**

A total of 52,723 participants without gallstone disease at baseline were included. Participants were stratified into five groups according to METS-IR quintiles. Multivariable Cox proportional hazards regression models were used to assess the association between METS-IR and the risk of developing gallstone disease. Restricted cubic spline (RCS) was employed to examine the dose-response relationship. Threshold effect analysis was subsequently conducted to identify potential inflection points. Additionally, subgroup and sensitivity analyses were performed to evaluate the robustness and reliability of the findings.

**Results:**

Over a mean follow-up of 3.13 years, 1,407 incident cases of gallstone disease occurred. Compared to the Q2 group, the risk was significantly higher in Q3 (hazard ratio (HR)=1.29, 95% confidence interval (CI): 1.08–1.54), Q4 (HR = 1.50, 95% CI: 1.25–1.80), and Q5 (HR = 1.85, 95% CI: 1.53–2.25), but not in Q1 (HR = 1.12, 95% CI: 0.93–1.35). A nonlinear U-shaped association was identified, with an inflection point at a METS-IR of 29.83. Below this point, each unit increase in METS-IR was associated with a 2.7% lower risk (HR = 0.973, 95% CI: 0.956–0.991). Above it, each unit increase was associated with a 4.1% higher risk (HR = 1.041, 95% CI: 1.031–1.051). Subgroup and sensitivity analyses confirmed robustness.

**Conclusions:**

The METS-IR exhibits a nonlinear U-shaped association with the risk of incident gallstone disease. Both lower and higher METS-IR levels, relative to the identified inflection point, are associated with an elevated risk of incident gallstone disease.

## Introduction

Gallstones are solid deposits formed in the gallbladder or biliary tract due to abnormal elevations in cholesterol or bilirubin (a breakdown product of hemoglobin) in the bile (1). As a common digestive system disorder, gallstone disease represents a major global public health issue. Studies indicate that the worldwide prevalence of gallstone disease reaches 6.1% (2), with a rising trend in recent years, leading to an increasing number of affected individuals (3) and a gradual shift toward younger populations (4). Common complications of gallstone disease include cholecystitis, pancreatitis, and gallbladder cancer (5, 6). Evidence suggests that gallstone disease is not only an independent risk factor for cardiovascular diseases but also significantly increases the risk of non-alcoholic fatty liver disease (NAFLD) and renal cancer (7–9) imposing a substantial medical and economic burden on society. Therefore, early prevention and treatment of gallstone disease are of critical importance.

Risk factors for gallstone disease include advanced age, female sex, genetic predisposition, metabolic syndrome, insulin resistance (IR), and obesity (10, 11). Among these, IR is a major risk factor. IR refers to a pathophysiological condition characterized by reduced responsiveness of peripheral tissues to insulin, resulting from decreased insulin receptor number or binding capacity, or diminished insulin sensitivity (12). Previous study has clearly indicated that hepatic insulin resistance increases the risk of gallstone disease (13), and elevated levels of various surrogate indicators of IR are significantly associated with a higher risk of gallstone disease (14). The Metabolic Score for Insulin Resistance (METS-IR), recently proposed by Bello-Chavolla et al. (15), integrates multiple metabolic parameters, serving as a comprehensive tool for assessing IR and metabolic status. Researches have shown that METS-IR outperforms other IR-related indicators in predicting all-cause and cardiovascular mortality (16), type 2 diabetes (15), and chronic kidney disease (17).

To date, studies on the association between METS-IR and gallstone disease remain limited to cross-sectional designs, and most rely on questionnaire-based diagnoses of gallstone disease, which may introduce information bias. Therefore, based on data from a health examination cohort, this study employs abdominal ultrasound for the diagnosis of gallstone disease. It aims to thoroughly investigate the association between METS-IR and incident gallstone disease, addressing gaps in the current literature and providing more robust epidemiological evidence regarding this association.

## Methods

### Study population

**Figure 1:**
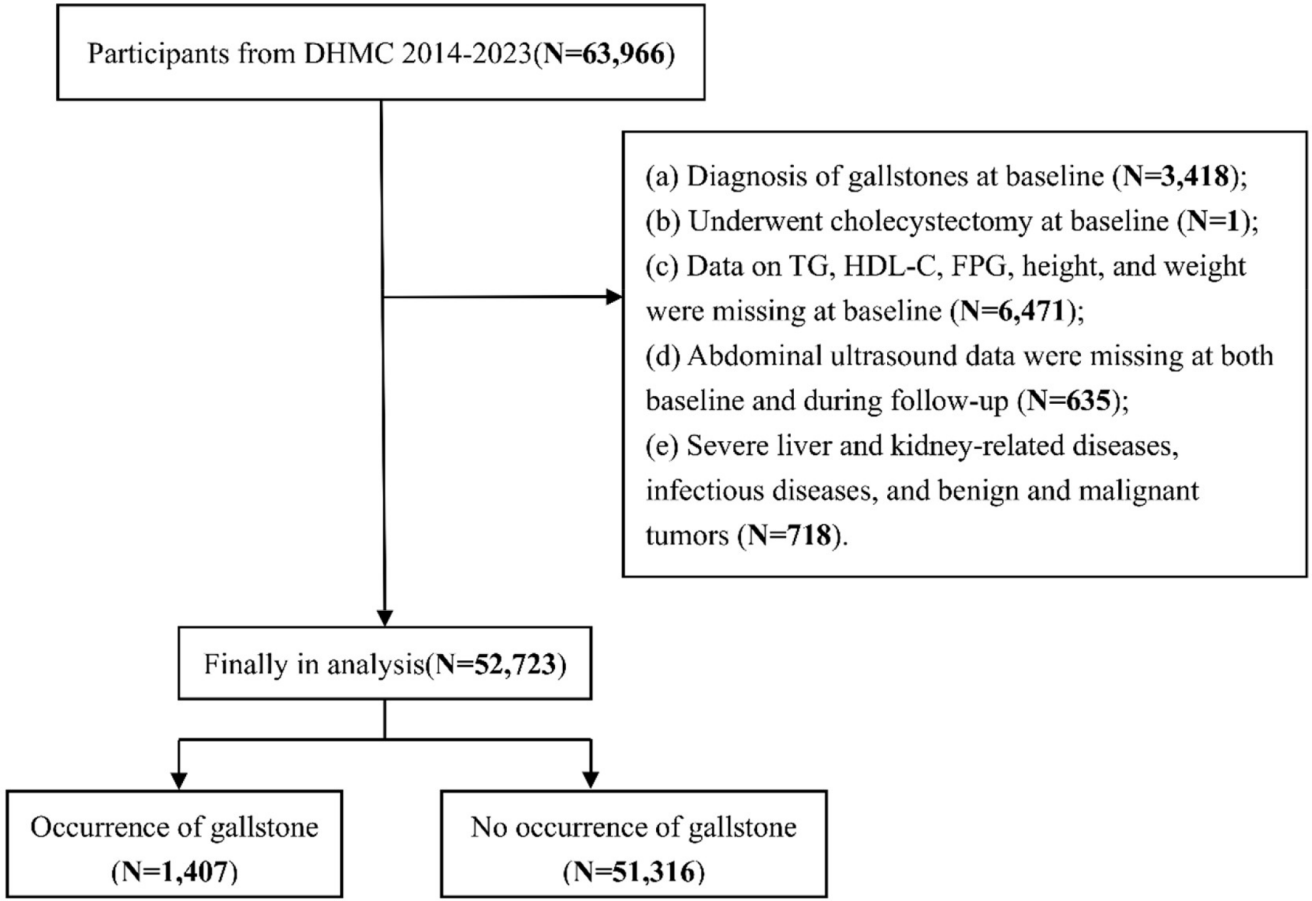
The flowchart of this study.

The data for this study were derived from the Dalian Health Management Cohort (DHMC; Cohort Number: CCC2023112102). Initiated in 2014, the DHMC is a large cohort study conducted at the Second Affiliated Hospital of Dalian Medical University of China, which collects physical examination data to assess population health status and facilitate health management. A total of 63,966 participants aged 18 years or older who underwent at least two health examinations between 2014 and 2023 were initially included. The first examination was defined as the baseline. Participants were followed from baseline until their last available examination or the diagnosis of gallstone disease, whichever occurred first. The exclusion criteria were as follows: (a) presence of gallstone disease at baseline (N = 3,418); (b) history of cholecystectomy at baseline (N = 1); (c) missing data on triglycerides, high-density lipoprotein cholesterol, fasting blood glucose, height, or weight at baseline (N = 6,471); (d) missing abdominal ultrasound data at baseline or during follow-up (N = 635); and (e) severe hepatic or renal disease, active infection, or malignancy (N = 718). After exclusions, 52,723 participants were included in the final analysis. **Figure 1** illustrates the participant selection process. This study was conducted in accordance with the principles of the Declaration of Helsinki and was approved by the Ethics Review Committee of the Second Affiliated Hospital of Dalian Medical University (Approval No.: KY2025-652-01-01). The Ethics Committee waived the requirement for informed consent.

### Data collection and definitions

Demographic information, including sex, age, medical history, and medication use, was collected using standardized self-administered questionnaires. Height and body weight were measured by trained healthcare professionals with participants in lightweight clothing and without shoes. Body mass index (BMI) was calculated as weight in kilograms divided by height in meters squared (kg/m²). Waist circumference (WC) was measured by trained staff using a soft tape measure. For blood pressure measurement, participants were asked to refrain from physical activity and to rest in a seated position for at least 5 minutes. Systolic and diastolic blood pressures were then measured three times at 5-minute intervals using an Omron electronic blood pressure monitor (HBP-9020, Japan), and the average of the three readings was recorded as the final value. Fasting venous blood samples were collected after an 8-hour fast. White blood cell count (WBC), red blood cell count (RBC), absolute neutrophil count (ANC), absolute monocyte count (AMC), absolute lymphocyte count (ALC), hemoglobin (HB), platelet count (PLT), total protein(TP), albumin (ALB), alanine aminotransferase (ALT), aspartate aminotransferase (AST), triglycerides (TG), total cholesterol (TC), high-density lipoprotein cholesterol (HDL-C), low-density lipoprotein cholesterol (LDL-C), serum uric acid (SUA), blood urea nitrogen (BUN), creatinine (Scr), fasting plasma glucose (FPG), lactate dehydrogenase (LDH), alkaline phosphatase (ALP), gamma-glutamyl transferase (GGT), total bilirubin (TBil), and direct bilirubin (DBil) were analyzed using a Roche Cobas c 501 chemistry analyzer under standard laboratory conditions.

Hypertension was defined as a self-reported history of hypertension, current use of antihypertensive medication, systolic blood pressure (SBP) ≥140 mmHg, or diastolic blood pressure (DBP) ≥90 mmHg (18). Diabetes mellitus was defined as the use of glucose-lowering drugs or insulin, a self-reported history of diabetes, FPG ≥7.0 mmol/L, or HbA1c ≥6.5% (19). Dyslipidemia was defined as the use of lipid-lowering medication, a self-reported history of dyslipidemia, or the presence of any of the following: TG ≥200 mg/dL, TC ≥240 mg/dL, LDL-C ≥160 mg/dL, or HDL-C ≤40 mg/dL (20).

### METS-IR and gallstone disease definitions

METS-IR was calculated using the following formula [15]: ln[2 × FPG (mg/dL) + TG (mg/dL)] × BMI (kg/m²)/ln[HDL-C (mg/dL)] (15). Gallstone disease was diagnosed using abdominal ultrasonography as part of routine health examinations conducted at the Health Management Center. All examinations were performed according to standardized clinical protocols by trained and experienced sonographers. Although ultrasound equipment may have undergone routine upgrades during the study period, standardized operating procedures and uniform diagnostic criteria were consistently applied. In this study, gallstone disease was defined as the presence of gallbladder or biliary stones detected on abdominal ultrasonography (21).

### Statistical analysis

Continuous variables with normal distribution are presented as mean ± standard deviation, while those with non-normal distribution are expressed as median and interquartile range. Differences between groups were assessed using one-way analysis of variance (ANOVA) or the Kruskal-Wallis H test, as appropriate. Categorical variables are summarized as numbers (percentages) and compared using the chi-square test. To maintain statistical power and minimize bias, missing values in covariates were handled using the k-nearest neighbors (KNN) imputation algorithm. For descriptive purposes, participants were categorized into five groups according to quintiles of baseline METS-IR levels: Q1 (METS-IR ≤29.05), Q2 (29.05 < METS-IR ≤33.44), Q3 (33.44 < METS-IR ≤37.69), Q4 (37.69 < METS-IR ≤42.81), and Q5 (METS-IR >42.81). Baseline characteristics were described according to gallstone disease status and METS-IR quintiles. Multivariable Cox proportional hazards regression models were used to evaluate the association between METS-IR and the risk of gallstone disease. Three models were constructed: Model 1 was unadjusted; Model 2 was adjusted for sex and age; and Model 3 was further adjusted for hypertension, diabetes, and dyslipidemia. Results are expressed as hazard ratio (HR) with 95% confidence interval (CI). The selection of covariates was based on prior epidemiological evidence and clinical relevance. Age and sex were considered potential confounders because they are associated with both IR and the risk of gallstone disease. Diabetes, hypertension, and dyslipidemia were further included as clinically relevant metabolic comorbidities related to metabolic dysfunction and gallstone formation. All covariates were assessed for multicollinearity using variance inflation factors, and no significant multicollinearity was detected (**Supplementary Table 1**). Sequential adjustment models were constructed to evaluate the stability of the association between METS-IR and gallstone disease under different levels of covariate control. Cumulative incidence of gallstone disease across METS-IR quintiles was illustrated using Kaplan-Meier curves, and differences were compared using the log-rank test. As the primary analytical approach, a restricted cubic spline (RCS) model with four knots was fitted within the Cox regression framework to explore the dose-response relationship between METS-IR with incident gallstone disease, adjusting for covariates in Model 3. After confirming a nonlinear association, threshold effect analysis was performed to identify potential inflection points. Subgroup analyses were conducted by sex, age, diabetes, hypertension, and dyslipidemia to examine potential effect modification. Three sensitivity analyses were performed to assess the robustness of the findings: first, participants who developed gallstone disease within the first year of follow-up were excluded to minimize potential reverse causality; second, the primary analyses were repeated using the original dataset without imputation; finally, WC was further adjusted for on the basis of Model 3 to examine the stability of the observed U-shaped association. All statistical analyses were performed using Stata version 17.0 and R version 4.3.2. A two-sided *p*-value <0.05 was considered statistically significant.

## Results

### Baseline characteristics

This cohort study included 52,723 participants, among whom 1,407 developed gallstone disease during a mean follow-up of 3.13 ± 1.92 years. The median age at baseline was 42 years (IQR: 32–53), and 53.22% of participants were male. Compared to those who did not develop gallstone disease, participants who developed incident gallstone disease had a significantly higher prevalence of hypertension, dyslipidemia, and diabetes (*P* < 0.05). They also exhibited higher WC, BMI, SBP, DBP, FPG, TC, LDL-C, and METS-IR levels (*P* < 0.05). However, no significant differences were observed in sex distribution and TG level (*P* > 0.05). Detailed baseline data are presented in **Table 1**. To improve clarity and readability, several less critical laboratory variables have been moved to the **Supplementary Material** (**Supplementary Table 2**). Furthermore, **Supplementary Table 3** presents the baseline characteristics stratified by METS-IR quintiles. Several baseline variables differed across the quintile groups. The incidence of gallstone disease across the five METS-IR groups was 2.14%, 2.04%, 2.68%, 3.07%, and 3.40%, respectively. Using the Q2 group (lowest incidence) as the reference, the absolute risk differences were +0.10%, +0.64%, +1.03%, and +1.36% for Q1, Q3, Q4, and Q5, respectively.

**Table 1:** Baseline characteristics of the population grouped by incident gallstone disease status during follow-up (N = 52,723).

Variable	Overall N = 52,723	Yes N = 1,407	No N = 51,316	*P*-value
AGE, years	42.0 (32.0, 53.0)	46.0 (35.0, 56.0)	42.0 (32.0, 53.0)	<0.001
SEX, n (%)				0.076
Female	24,666 (46.78%)	625 (44.42%)	24,041 (46.85%)	
Male	28,057 (53.22%)	782 (55.58%)	27,275 (53.15%)	
WC, cm	84.27 ± 10.82	86.71 ± 10.82	84.21 ± 10.81	<0.001
BMI, kg/m^2^	24.02 ± 3.98	24.79 ± 4.16	24.00 ± 3.97	<0.001
TC, mmol/L	4.93 ± 0.91	5.00 ± 0.94	4.93 ± 0.91	0.002
TG, mmol/L	1.4 (1.0, 2.0)	1.4 (1.0, 2.0)	1.4 (1.0, 2.0)	0.127
HDL-C, mmol/L	1.34 ± 0.32	1.30 ± 0.32	1.34 ± 0.32	<0.001
LDL-C, mmol/L	2.66 ± 0.73	2.72 ± 0.76	2.66 ± 0.73	0.003
FPG, mmol/L	5.55 ± 0.60	5.59 ± 0.63	5.55 ± 0.60	0.005
SBP, mmHg	127.55 ± 16.93	129.85 ± 18.41	127.49 ± 16.88	<0.001
DBP, mmHg	76.95 ± 11.24	78.12 ± 11.88	76.92 ± 11.22	<0.001
Hypertension, n(%)				<0.001
no	40,191 (76.23%)	989 (70.29%)	39,202 (76.39%)	
yes	12,532 (23.77%)	418 (29.71%)	12,114 (23.61%)	
Dyslipidemia, n(%)				0.002
no	35,051 (66.48%)	880 (62.54%)	34,171 (66.59%)	
yes	17,672 (33.52%)	527 (37.46%)	17,145 (33.41%)	
Diabetes, n(%)				0.007
no	48,773 (92.51%)	1,275 (90.62%)	47,498 (92.56%)	
yes	3,950 (7.49%)	132 (9.38%)	3,818 (7.44%)	
METS-IR	35.54 (30.2, 41.31)	37.22 (32.13, 43.03)	35.49 (30.15, 41.26)	<0.001

WC, Waist Circumference; BMI, Body Mass Index; TC, Total Cholesterol; TG, Triglycerides; HDL-C, High-Density Lipoprotein Cholesterol; LDL-C, Low-Density Lipoprotein Cholesterol; FPG, Fasting Plasma Glucose; SBP, Systolic Blood Pressure; DBP, Diastolic Blood Pressure; METS-IR, the Metabolic Score for Insulin Resistance.

### Association between METS-IR levels and incident gallstone disease

**Figure 2:**
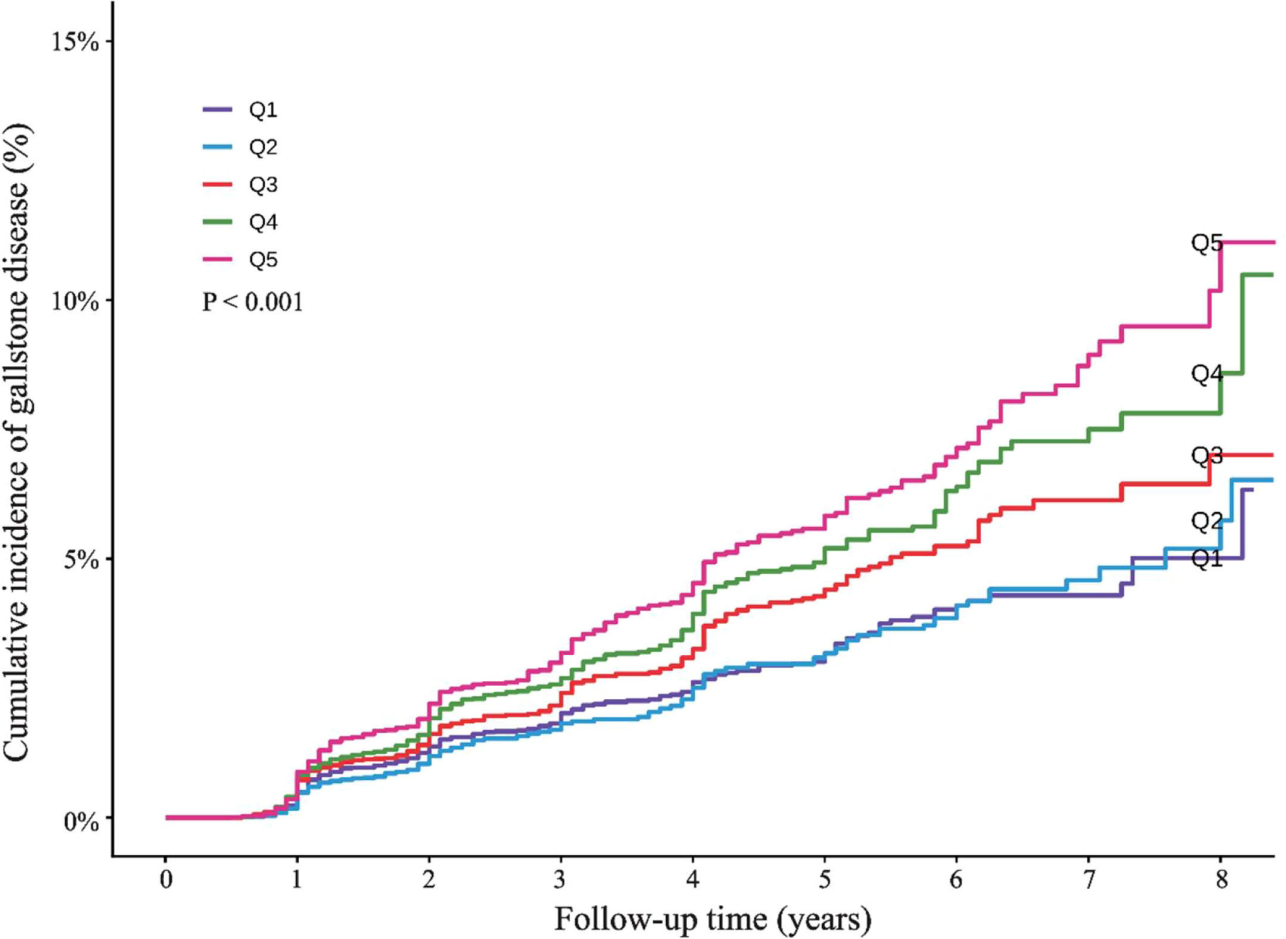
Comparison of cumulative incidence of gallstone disease in people with different quintiles of METS-IR levels. METS-IR, the Metabolic Score for Insulin Resistance.

Cox proportional hazards models were used to examine the association between METS-IR and gallstone disease (**Table 2**). In the unadjusted model, analyzed as a continuous variable, each 1-unit increment in METS-IR was associated with a 3% increase in the risk of gallstone disease (HR = 1.03, 95% CI: 1.02–1.03, *P* < 0.001). This positive association remained significant in the fully adjusted Model 3 (HR = 1.02, 95% CI: 1.02–1.03, *P* < 0.001). When METS-IR was categorized into quintiles, with the second quintile (Q2) as the reference (representing the lowest risk in the Kaplan-Meier analysis), the fully adjusted Model 3 revealed a graded increase in risk across higher quintiles. Specifically, compared with Q2, the risk was significantly elevated in Q3 (HR = 1.29, 95% CI: 1.08–1.54, *P* = 0.005), Q4 (HR = 1.50, 95% CI: 1.25–1.80, *P* < 0.001), and Q5 (HR = 1.85, 95% CI: 1.53–2.25, *P* < 0.001). No statistically significant association was observed for the lowest quintile (Q1) (HR = 1.12, 95% CI: 0.93–1.35, *P* = 0.236). Consistent with these findings, the Kaplan-Meier curve (**Figure 2**) demonstrated a significant divergence in the cumulative incidence of gallstone disease across METS-IR quintiles (log-rank *P* < 0.001). The cumulative incidence was lowest in Q2 and highest in Q5.

**Table 2:** Association of METS-IR overall and quintile group levels with incident gallstone disease.

Variable	Model 1		Model 2		Model 3	
	HR (95%CI)	*P* value	HR (95%CI)	*P* value	HR (95%CI)	*P* value
METS-IR	1.03(1.02, 1.03)	<0.001	1.02(1.02, 1.03)	<0.001	1.02(1.02, 1.03)	<0.001
Q1	1.04(0.87, 1.26)	0.646	1.12(0.93, 1.35)	0.238	1.12(0.93, 1.35)	0.236
Q2	Reference		Reference		Reference	
Q3	1.36(1.14, 1.62)	<0.001	1.28(1.07, 1.54)	0.006	1.29(1.08, 1.54)	0.005
Q4	1.60(1.34, 1.90)	<0.001	1.48(1.24, 1.76)	<0.001	1.50(1.25, 1.80)	<0.001
Q5	1.86(1.57, 2.20)	<0.001	1.78(1.49, 2.13)	<0.001	1.85(1.53, 2.25)	<0.001

Model 1 was unadjusted, and Model 2 was adjusted for Sex and Age. Model 3 added other covariates to Model 2, including Diabetes, Hypertension, and Dyslipidemia.

HR, Hazard Ratio; CI, Confidence Interval; METS-IR, the Metabolic Score for Insulin Resistance.

### Nonlinear association and threshold effect analysis

**Figure 3:**
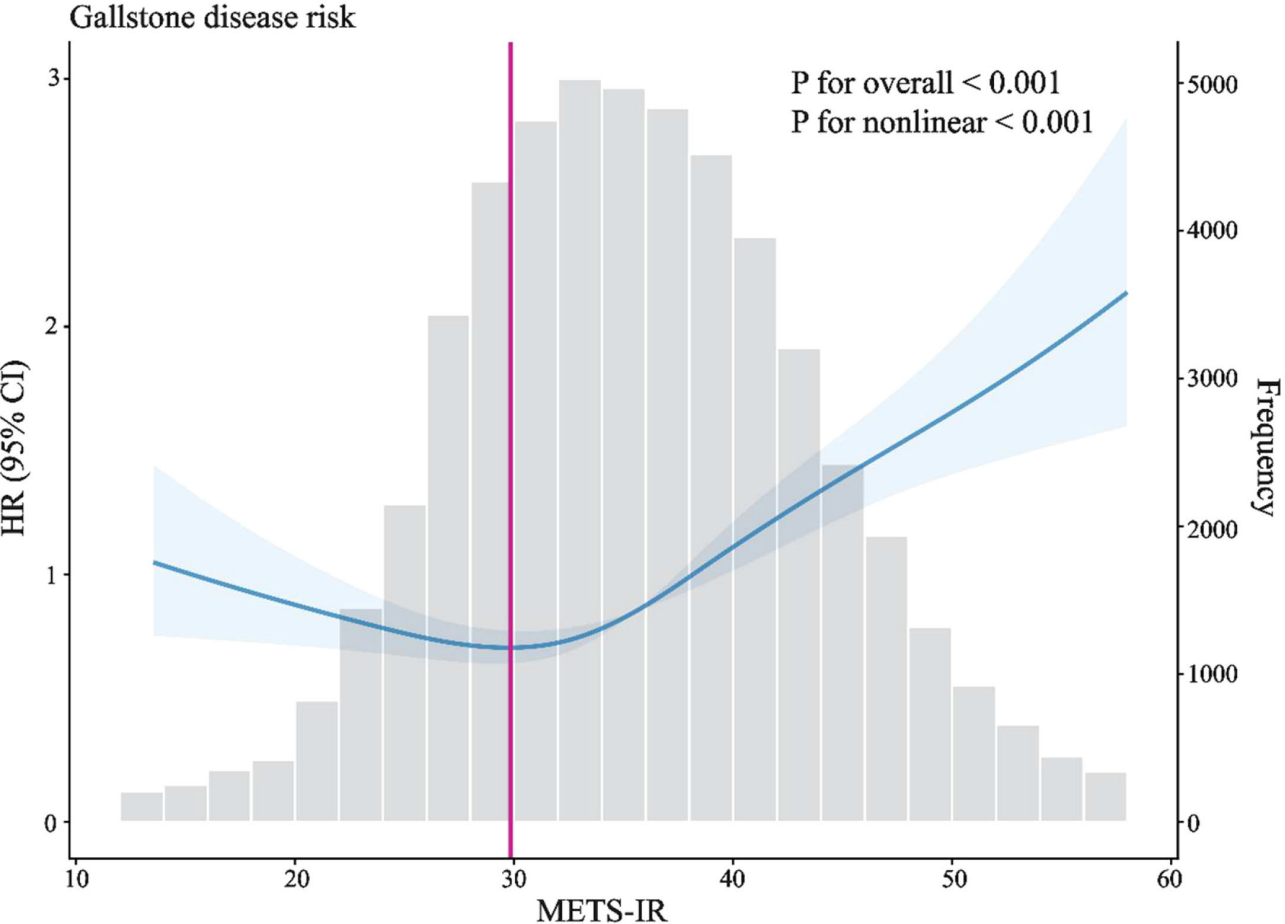
Dose-response relationship between METS-IR and incident gallstone disease. Adjusted for Sex, Age, Diabetes, Hypertension, and Dyslipidemia. Solid lines represent the estimates, and the shaded blue areas indicate their corresponding 95% confidence intervals (CIs). The grey histogram indicates the frequency distribution of METS-IR values in the study population. HR, Hazard Ratio; CI, Confidence Interval; METS-IR, the Metabolic Score for Insulin Resistance.

Restricted cubic spline analysis revealed a U-shaped association between METS-IR and gallstone disease (*P* for overall <0.001, *P* for nonlinear <0.001; **Figure 3**) and this pattern remained consistent across different sex populations (**Supplementary Figure 1**). Subsequent threshold effect analysis, using a two-piecewise Cox proportional hazards model (likelihood ratio test *P* < 0.001), identified an inflection point at a METS-IR value of 29.83. Below this threshold, each 1-unit increase in METS-IR was associated with a 2.7% decrease in gallstone disease risk (HR = 0.973, 95% CI: 0.956–0.991, *P* = 0.003). Conversely, above this threshold, each 1-unit increase in METS-IR was associated with a 4.1% increase in risk (HR = 1.041, 95% CI: 1.031–1.051, *P* < 0.001). In other words, the risk of incident gallstone disease decreased as METS-IR increased up to approximately 29.83 and increased thereafter. Detailed results are shown in **Table 3**.

**Table 3:** Threshold effect analysis of METS-IR on incident gallstone disease.

Outcome	HR (95%CI)	*P* value
Fitting by standard linear model	1.024(1.016, 1.031)	**<0.001**
Fitting by the two-piecewise Cox proportional risk model		
Inflection point	29.83	
< 29.83	0.973(0.956, 0.991)	**0.003**
> 29.83	1.041(1.031, 1.051)	**<0.001**
*P* for likelihood ratio test		**<0.001**

Adjusted for Sex, Age, Diabetes, Hypertension, and Dyslipidemia.

HR, Hazard Ratio; CI, Confidence Interval; METS-IR, the Metabolic Score for Insulin Resistance.

Bold values indicate statistical significance (P < 0.05).

### Subgroup analysis

**Figure 4:**
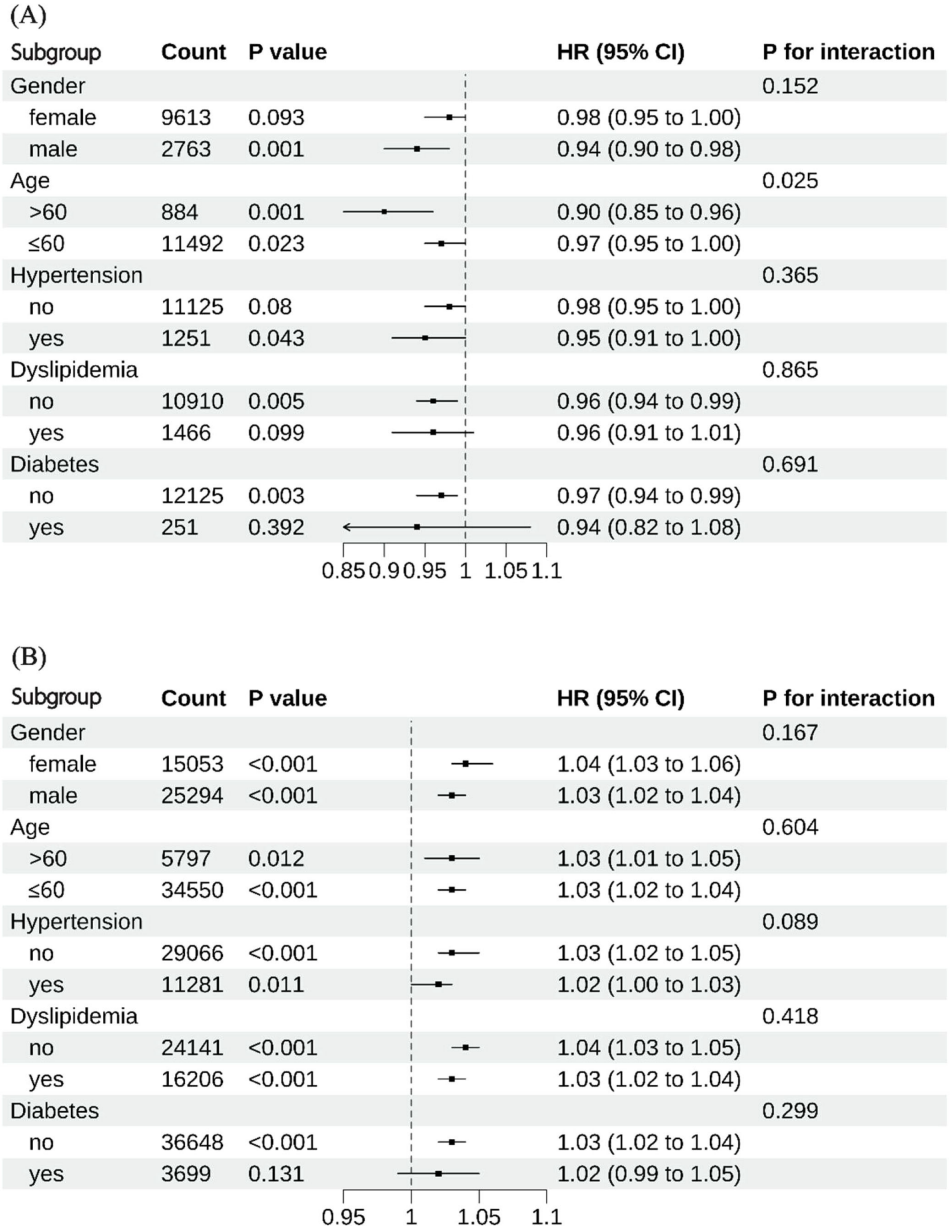
Subgroup analysis of the association between METS-IR and incident gallstone disease. **(A)** METS-IR < 29.83; **(B)** METS-IR ≥ 29.83. HR, Hazard Ratio; CI, Confidence Interval; METS-IR, the Metabolic Score for Insulin Resistance.

Subgroup analyses were performed to assess the consistency of the U-shaped association across different populations (**Figure 4**). The association remained consistent in all subgroups stratified by sex, age, hypertension, diabetes, and dyslipidemia. Notably, age was identified as an effect modifier in the segment below the inflection point (METS-IR < 29.83). A more pronounced protective effect per unit increase in METS-IR was observed in participants aged >60 years (HR = 0.90, 95% CI: 0.85–0.96) compared to those aged ≤60 years (HR = 0.97, 95% CI: 0.95–1.00; *P* for interaction = 0.025).

### Sensitivity analyses

Three sensitivity analyses were conducted. In the first analysis, excluding participants who developed the outcome within one year of follow-up yielded consistent results: below the inflection point, each 1-unit increase in METS-IR was associated with a 3% risk reduction (HR = 0.97, 95% CI: 0.94–0.99, *P* = 0.011), whereas above the inflection point, each 1-unit increase was associated with a 4% risk increase (HR = 1.04, 95% CI: 1.03–1.05, *P* < 0.001). The second analysis, using the original dataset without imputation, also confirmed the stability and statistical significance of the U-shaped association (METS-IR < 29.83: HR = 0.97, 95% CI: 0.94–0.99, *P* = 0.004; METS-IR ≥ 29.83: HR = 1.04, 95% CI: 1.03–1.05, *P* < 0.001). The third analysis, additionally adjusted for WC based on Model 3, also yielded results consistent with the primary analysis (**Supplementary Table 4**).

## Discussion

In this large cohort study, after adjusting for multiple confounders, the nonlinear association between METS-IR and incident gallstone disease was primarily identified using continuous-variable modeling with restricted cubic splines, revealing a U-shaped association with an inflection point at a METS-IR value of 29.83. Specifically, the risk of incident gallstone disease decreased as METS-IR increased up to this threshold, whereas beyond this point the risk increased with further increases in METS-IR. This association remained consistent across various subgroups and in sensitivity analyses. Our findings indicate that the METS-IR index is independently associated with the risk of incident gallstone disease in adults, which may provide epidemiological insights into metabolic factors associated with gallstone disease.

IR, a component of metabolic syndrome, has been widely associated with gallstone formation (22). A prospective cohort study by Beyza N Aydin et al. found that hepatic insulin resistance was associated with a higher risk of gallstone disease among Native Americans in the Southwestern United States (13). Furthermore, several surrogate IR indices, including the Triglyceride-Glucose (TyG) index, Homeostatic Model Assessment of Insulin Resistance (HOMA-IR), and METS-IR, have been correlated with gallstone disease (23, 24). Previous research by Jin Wang et al., using the NHANES database, reported a linear positive association, with each unit increase in METS-IR corresponding to a 3.3% higher prevalence of gallstone disease (OR = 1.033, 95% CI: 1.0258–1.0403), restricted cubic spline fitting indicated that the dose–response relationship was nearly linear (25). Similarly, a cross-sectional study by Rui Gong et al. found comparable results (14). Our study found that, after full adjustment, each one-unit increase in METS-IR was associated with a 2.4% higher risk of gallstone disease (HR = 1.024; 95% CI: 1.016–1.031, *P* < 0.001). Subsequent RCS analysis, however, revealed a nonlinear U-shaped association, which was further confirmed by threshold effect analysis. Multiple factors may explain the differences between our findings and previous studies reporting a linear positive association. First, most previous studies were cross-sectional in design and relied on self-reported gallstone disease diagnoses, whereas our study employed a prospective cohort design and used abdominal ultrasonography to confirm incident gallstone disease. This approach reduces potential information bias and allows for a clearer assessment of temporal relationships. Second, differences in study populations may also contribute to the inconsistent findings. Prior analyses were primarily based on data from the U.S. NHANES database, whereas our cohort was derived from a Northern Chinese population. Variations in genetic background, dietary patterns, and lifestyle factors may influence the relationship between metabolic status and gallstone formation. Third, differences in statistical modeling strategies may partly explain the divergent results. Previous studies generally applied linear models, while our analysis incorporated RCS modeling and threshold effect analysis, which allowed us to detect a nonlinear U-shaped association between METS-IR and the risk of gallstone disease. Additionally, while Jin Wang et al. reported in subgroup analyses that the association between increasing METS-IR and gallstone disease prevalence was stronger in women than in men (25), we found no such interaction above the inflection point in our study (*P* for interaction = 0.167). Interestingly, below the inflection point, the protective association of increasing METS-IR was more pronounced among individuals aged >60 years (*P* for interaction = 0.025). This finding suggests that older adults who exhibit a low insulin-resistance state may paradoxically be more vulnerable to gallstone disease development. In elderly populations, a low insulin-resistance phenotype is often linked to sarcopenia, frailty, or other chronic disease, conditions known to impair gallbladder motility and increase the risk of gallstone disease (26). In contrast, studies have shown that among adults younger than 50 years, obesity and metabolic syndrome are the major determinants of gallstone formation (27). Therefore, younger individuals with low IR typically represent a genuinely healthy metabolic profile and consequently have a lower risk of gallstone disease compared with older adults.

The mechanisms underlying the association between METS-IR and gallstone disease are not fully elucidated, though the IR represented by METS-IR is considered pivotal. Studies suggest that IR may contribute to gallstone formation through mechanisms related to abnormal lipid metabolism and biliary system dysfunction (1). Visceral obesity and hepatic insulin resistance have also been implicated in promoting cholesterol supersaturation in bile, thereby facilitating gallstone formation (28). A review from Mexico indicated that higher plasma insulin levels stimulate the activity of HMG-CoA reductase, leading to cholesterol hypersecretion and the development of cholesterol gallstones (29). Furthermore, a genetic analysis suggested that type 2 diabetes, which is underpinned by IR, increases the risk of gallstone disease through the regulation of two genes: HNF4A, which modulates liver-specific gene expression involved in lipid transport, glucose, and bile acid metabolism (30), and GCKR, which enhances hepatic cholesterol availability, leading to increased biliary cholesterol concentration and gallstone formation (31). Contrary to previously reported linear associations, our study identified a U-shaped association between METS-IR and the risk of gallstone disease. While the increased risk observed at higher METS-IR levels is biologically plausible and consistent with mechanisms related to IR, the elevated risk observed at lower METS-IR levels remains poorly understood. One possible explanation is that individuals with very low METS-IR values may have lower BMI or potential undernutrition, which could influence gallbladder motility and bile composition. Gallbladder contraction is primarily stimulated by cholecystokinin (CCK) released after meals, and reduced food intake may impair gallbladder emptying, potentially leading to bile stasis and promoting cholesterol crystallization. In addition, underweight status or nutritional deficiencies may alter hepatic lipid metabolism and bile acid composition, which could contribute to relative cholesterol supersaturation despite lower systemic lipid levels. However, these explanations remain speculative, as our study did not include direct measures of nutritional status, weight-change history, dietary intake, or gallbladder motility. Future studies incorporating these factors are needed to clarify the biological mechanisms underlying the increased risk observed at lower METS-IR levels.

### Strengths and limitations

Key strengths of our study include its large cohort design, which for the first time reveals a nonlinear U-shaped association between METS-IR and incident gallstone disease. The robustness of this finding was reinforced by subgroup and sensitivity analyses. Furthermore, unlike previous studies relying on self-reported questionnaires for gallstone disease diagnosis, we used standardized abdominal ultrasonography, significantly reducing information bias. Several limitations should be acknowledged. First, all participants were recruited from a single health management center and consisted of voluntary health examination attendees from Northern China, which may limit the generalizability of our findings to other regions, ethnic groups, and healthcare settings. More importantly, recruiting participants through routine health examinations may introduce selection bias. Individuals who participate in regular health screening programs often differ systematically from the general population in terms of health awareness, socioeconomic status, and baseline comorbidity profiles. Such a selection process may influence the distribution of the METS-IR spectrum within the study population and potentially affect the observed shape of the association. For example, if individuals with relatively favorable metabolic status are disproportionately represented in the lower range of METS-IR, this could potentially contribute to the appearance of a U-shaped association. Therefore, although a U-shaped association was observed in our cohort, the specific parameters of the curve—particularly the inverse association observed below the inflection point—should be interpreted with caution. Future studies based on more population-representative cohorts are warranted to further validate these findings. Second, METS-IR was assessed only at baseline in this study because repeated measurements of metabolic parameters were not consistently available for all participants during follow-up. During the approximately three-year follow-up period, metabolic parameters, body weight, dietary habits, and physical activity levels may have changed. Treating METS-IR as a fixed exposure may therefore introduce time-varying exposure misclassification and lead to regression dilution bias, potentially attenuating the estimated associations, including the observed threshold and U-shaped association. Future studies incorporating repeated measurements of metabolic indicators would help clarify the impact of temporal changes in METS-IR on the risk of gallstone disease. Third, although we adjusted for multiple covariates, unmeasured factors, including dietary patterns, caloric intake, rapid weight change, physical activity, reproductive history, history of bariatric surgery, and related medication use (e.g., estrogen therapy or oral contraceptives), may still have introduced residual confounding. Therefore, the observed associations should be interpreted as observational relationships rather than causal effects. Future studies with more comprehensive covariate information and more representative prospective cohorts are needed to further validate these findings. Finally, although abdominal ultrasonography is a standard non-invasive method for diagnosing gallstone disease, its sensitivity for detecting microlithiasis or bile duct stones is limited. In addition, potential variability between ultrasound operators may introduce diagnostic bias.

## Conclusion

In summary, our study demonstrates a U-shaped association between the METS-IR index and the risk of incident gallstone disease, with an inflection point at 29.83. These findings suggest that METS-IR may serve as a potential marker for identifying individuals at higher risk of gallstone disease. However, further studies are required to determine whether METS-IR can be used to guide prevention or intervention strategies.

## Data Availability

The data analyzed in this study is subject to the following licenses/restrictions: The datasets used and/or analyzed during the current study are available from the corresponding author on reasonable request. Requests to access these datasets should be directed to Song Leng, dllengsong@163.com.
