# Supplementary material for: The U-shaped association between the metabolic score for insulin resistance and incident gallstone disease: a population-based cohort study
Source: Front Endocrinol (Lausanne). 2026 Mar 27;17:1804048. doi: 10.3389/fendo.2026.1804048 (PMC13065699; doi:10.3389/fendo.2026.1804048)
Supplement: Supplementary file 1 [file DataSheet1.docx]

**The U-Shaped Association Between the Metabolic Score for Insulin Resistance and Incident Gallstone Disease: A Population-Based Cohort Study**

Yunxiang Ming ^a,b^, Yangxuan He ^a,b^, Jiayi Deng ^a,b^, Fei Xu a, Hong Chen ^a^, Yilin Zhu ^a^, Jingshan Jiang ^a^, Yang Liu ^a^ Song Leng ^a*^

**Supplementary Figure 1.** RCS analysis of the association between METS-IR and incident gallstone disease in female (**A**) and male (**B**) participants.

**Supplementary Table 1.** Variance Inflation Factor of Covariates

**Supplementary Table 2.** Supplementary Table 2. Additional baseline laboratory characteristics of participants according to incident gallstone disease status (N=52,723).

**Supplementary Table 3.** The baseline characteristics of the population grouped by METS-IR quintiles in the analysis of gallstone disease incidence rates (N=52,723).

**Supplementary Table 4.** Sensitivity analyses of the association between METS-IR index and gallstone disease.

**
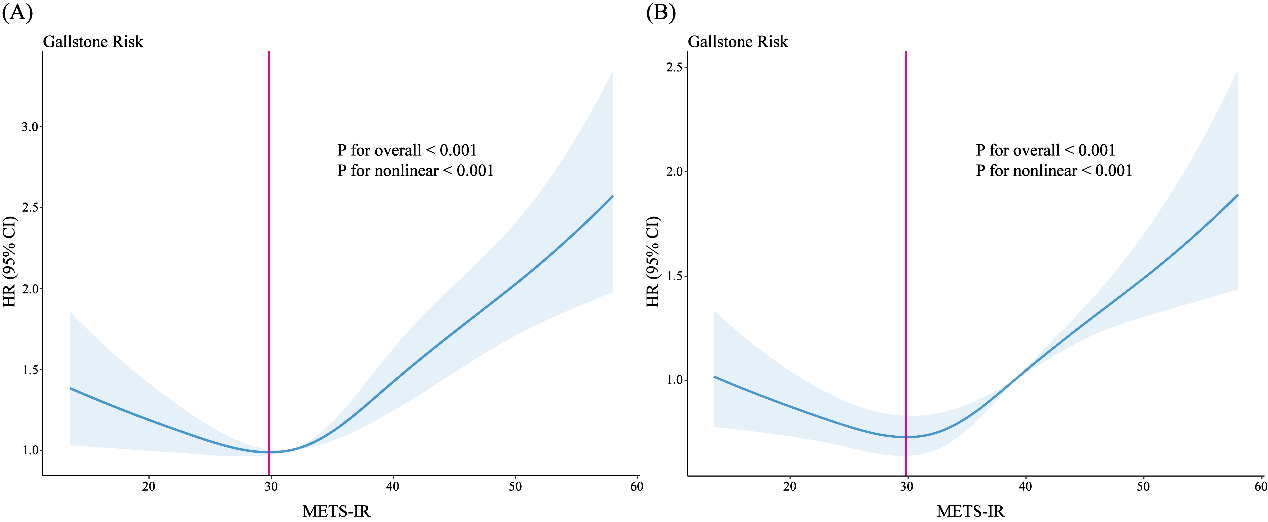
**

**Supplementary Figure 1.** RCS analysis of the association between METS-IR and incident gallstone disease in female (**A**) and male (**B**) participants.

Adjusted for Sex, Age, Diabetes, Hypertension, and Dyslipidemia. Solid lines represent the estimates, and the shaded blue areas indicate their corresponding 95% confidence intervals (CIs).

Abbreviations: HR, Hazard Ratio; CI, Confidence Interval; METS-IR, the Metabolic Score for Insulin Resistance; RCS, Restricted Cubic Spline.

**Supplementary Table 1.** Variance Inflation Factor of Covariates

| Variable | VIF | |
| --- | --- | --- |
|  | Model 3 | Sensitivity analyses 3 |
| METS-IR | 1.44 | 2.37 |
| Age | 1.26 | 1.30 |
| Sex | 1.21 | 1.49 |
| Hypertension | 1.28 | 1.30 |
| Diabetes | 1.12 | 1.27 |
| Dyslipidemia | 1.26 | 1.26 |
| WC |  | 2.91 |

Abbreviations: METS-IR, the Metabolic Score for Insulin Resistance; WC, Waist Circumference; VIF, Variance Inflation Factor.

**Supplementary Table 2. Additional baseline laboratory characteristics of participants according to incident gallstone disease status (N=52,723).**

| **Variable** | **Overall**  N = 52,723 | **Yes**  N = 1,407 | **No**  N = 51,316 | ***P*-value** |
| --- | --- | --- | --- | --- |
| **WBC, 10^9/L** | 6.13 ± 1.48 | 6.28 ± 1.53 | 6.13 ± 1.47 | <0.001 |
| **RBC, 10^9/L** | 4.83 ± 0.45 | 4.83 ± 0.45 | 4.83 ± 0.45 | 0.832 |
| **HB, g/L** | 146.0 (134.0, 157.0) | 146.0 (134.0, 157.0) | 146.0 (134.0, 157.0) | 0.222 |
| **PLT, 10^9/L** | 238.62 ± 52.54 | 234.50 ± 55.07 | 238.73 ± 52.46 | 0.003 |
| **ANC, 10^9/L** | 3.57 ± 1.10 | 3.66 ± 1.14 | 3.57 ± 1.10 | 0.003 |
| **ALC, 10^9/L** | 2.06 ± 0.56 | 2.10 ± 0.58 | 2.06 ± 0.56 | 0.006 |
| **AMC, 10^9/L** | 0.33 ± 0.11 | 0.35 ± 0.11 | 0.33 ± 0.11 | <0.001 |
| **SUA, μmol/L** | 348.80 ± 93.67 | 355.68 ± 95.04 | 348.61 ± 93.63 | 0.005 |
| **Scr, μmol/L** | 66.4 (56.0, 77.7) | 66.6 (56.1, 77.9) | 66.4 (55.9, 77.7) | 0.394 |

Abbreviations: WBC, White Blood Cell; RBC, Red Blood Cell; HB, Hemoglobin; PLT, Platelets; ANC, Absolute Neutrophil Count; ALC, Absolute Lymphocyte Count; AMC, Absolute Monocyte Count; SUA, Serum Uric Acid; Scr, Serum Creatinine METS-IR, the Metabolic Score for Insulin Resistance.

**Supplementary Table 3.** The baseline characteristics of the population grouped by METS-IR quintiles in the analysis of gallstone disease incidence rates (N=52,723).

| **Variable** | **Overall**  n = 52,723 | **Q1**  n = 10,545 | **Q2**  n = 10,544 | **Q3**  n = 10,545 | **Q4**  n = 10,544 | **Q5**  n =10,545 | ***P*-value** |
| --- | --- | --- | --- | --- | --- | --- | --- |
| **AGE, years** | 42.0 (32.0, 53.0) | 34.0 (28.0, 46.0) | 40.0 (31.0, 51.0) | 45.0 (34.0, 55.0) | 47.0 (35.0, 56.0) | 45.0(35.0,54.0) | <0.001 |
| **SEX, n (%)** |  |  |  |  |  |  | <0.001 |
| Female | 24,666 (46.78%) | 8,216 (77.91%) | 6,942 (65.84%) | 4,600 (43.62%) | 3,024 (28.68%) | 1,884(17.87%) |  |
| Male | 28,057 (53.22%) | 2,329 (22.09%) | 3,602 (34.16%) | 5,945 (56.38%) | 7,520 (71.32%) | 8,661(82.13%) |  |
| **WC, cm** | 84.27 ± 10.82 | 73.01 ± 8.10 | 78.00 ± 6.06 | 84.04 ± 6.09 | 89.44 ± 6.08 | 96.87 ± 7.54 | <0.001 |
| **BMI, kg/m^2^** | 24.02 ± 3.98 | 18.69 ± 2.50 | 22.18 ± 1.23 | 24.13 ± 1.35 | 26.04 ± 1.57 | 29.06 ± 2.44 | <0.001 |
| **WBC, 10^9/L** | 6.13 ± 1.48 | 5.68 ± 1.43 | 5.77 ± 1.36 | 6.05 ± 1.39 | 6.35 ± 1.41 | 6.82 ± 1.48 | <0.001 |
| **RBC**, **10^9/L** | 4.83 ± 0.45 | 4.58 ± 0.39 | 4.66 ± 0.41 | 4.83 ± 0.43 | 4.97 ± 0.42 | 5.09 ± 0.41 | <0.001 |
| **HB, g/L** | 146.0 (134.0, 157.0) | 135.0 (128.0, 144.0) | 138.0 (130.0, 149.0) | 146.0 (135.0, 157.0) | 152.0 (141.0, 160.0) | 156.0 (147.0, 163.0) | <0.001 |
| **PLT, 10^9/L** | 238.62 ± 52.54 | 240.38 ± 51.60 | 241.36 ± 51.70 | 238.00 ± 53.16 | 236.52 ± 52.57 | 236.83 ± 53.46 | 0.008 |
| **ANC, 10^9/L** | 3.57 ± 1.10 | 3.31 ± 1.10 | 3.36 ± 1.05 | 3.51 ± 1.05 | 3.71 ± 1.06 | 3.98 ± 1.10 | <0.001 |
| **ALC, 10^9/L** | 2.06 ± 0.56 | 1.92 ± 0.52 | 1.95 ± 0.51 | 2.04 ± 0.54 | 2.12 ± 0.55 | 2.28 ± 0.58 | <0.001 |
| **AMC, 10^9/L** | 0.33 ± 0.11 | 0.31 ± 0.11 | 0.31 ± 0.10 | 0.33 ± 0.11 | 0.34 ± 0.11 | 0.37 ± 0.12 | <0.001 |
| **SUA, μmol/L** | 348.80 ± 93.67 | 291.21 ± 74.39 | 309.09 ± 77.38 | 348.57 ± 82.37 | 379.61 ± 85.35 | 415.52 ± 89.37 | <0.001 |
| **Scr, μmol/L** | 66.4 (56.0, 77.7) | 57.7 (51.9, 66.3) | 60.5 (53.1, 72.1) | 68.0 (57.0, 78.5) | 72.2 (61.5, 80.8) | 74.0(64.6,82.0) | <0.001 |
| **TC, mmol/L** | 4.93 ± 0.91 | 4.77 ± 0.88 | 4.83 ± 0.91 | 4.95 ± 0.91 | 5.03 ± 0.91 | 5.06 ± 0.92 | <0.001 |
| **TG, mmol/L** | 1.4 (1.0, 2.0) | 0.9 (0.7, 1.3) | 1.1 (0.9, 1.5) | 1.4 (1.0, 1.8) | 1.7 (1.3, 2.2) | 2.3 (1.6, 3.1) | <0.001 |
| **HDL-C, mmol/L** | 1.34 ± 0.32 | 1.63 ± 0.32 | 1.48 ± 0.25 | 1.34 ± 0.23 | 1.21 ± 0.21 | 1.04 ± 0.21 | <0.001 |
| **LDL-C, mmol/L** | 2.66 ± 0.73 | 2.37 ± 0.68 | 2.57 ± 0.71 | 2.76 ± 0.71 | 2.85 ± 0.71 | 2.77 ± 0.75 | <0.001 |
| **FPG, mmol/L** | 5.55 ± 0.60 | 5.25 ± 0.49 | 5.35 ± 0.47 | 5.54 ± 0.53 | 5.70 ± 0.59 | 5.90 ± 0.65 | <0.001 |
| **SBP, mmHg** | 127.55 ± 16.93 | 119.90 ± 15.17 | 122.54 ± 15.30 | 127.91 ± 16.30 | 131.94 ± 16.49 | 135.45 ± 16.29 | <0.001 |
| **DBP, mmHg** | 76.95 ± 11.24 | 72.45 ± 10.02 | 73.23 ± 9.88 | 76.62 ± 10.68 | 79.61 ± 10.88 | 82.84 ± 11.18 | <0.001 |
| **Hypertension, n(%)** | 12,532 (23.77%) | 1,077 (10.21%) | 1,323 (12.55%) | 2,388 (22.65%) | 3,316 (31.45%) | 4,428(41.99%) | <0.001 |
| **Dyslipidemia, n(%)** | 17,672 (33.52%) | 1,285 (12.19%) | 1,403 (13.31%) | 2,548 (24.16%) | 4,697 (44.55%) | 7,739(73.39%) | <0.001 |
| **Diabetes, n(%)** | 3,950 (7.49%) | 232 (2.20%) | 226 (2.14%) | 518 (4.91%) | 1,039 (9.85%) | 1,935(18.35%) | <0.001 |
| **METS-IR** | 35.54 (30.2, 41.31) | 26.2 (23.72, 27.74) | 31.3 (30.2, 32.38) | 35.54 (34.47, 36.6) | 40.02 (38.82, 41.31) | 46.95 (44.59, 50.7) | <0.001 |
| **Gallstone, n (%)** | 1,407 (2.67%) | 226 (2.14%) | 215 (2.04%) | 283 (2.68%) | 324 (3.07%) | 359 (3.40%) | <0.001 |

Abbreviations: WC, Waist Circumference; BMI, Body Mass Index; WBC, White Blood Cell; RBC, Red Blood Cell; HB, Hemoglobin; PLT, Platelets; ANC, Absolute Neutrophil Count; ALC, Absolute Lymphocyte Count; AMC, Absolute Monocyte Count; SUA, Serum Uric Acid; Scr, Serum Creatinine; TC, Total Cholesterol; TG, Triglycerides; HDL-C, High-Density Lipoprotein Cholesterol; LDL-C, Low-Density Lipoprotein Cholesterol; FPG, Fasting Plasma Glucose; SBP, Systolic Blood Pressure; DBP, Diastolic Blood Pressure; METS-IR, the Metabolic Score for Insulin Resistance.

**Supplementary Table 4.** Sensitivity analyses of the association between METS-IR index and gallstone disease.

| **METS-IR index**  **(per unit increase)** | **Model 1** | | **Model 2** | | **Model 3** | |
| --- | --- | --- | --- | --- | --- | --- |
|  | ***HR* (95% CI)** | ***P* value** | ***HR* (95% CI)** | ***P* value** | ***HR* (95% CI)** | ***P* value** |
| **Sensitivity analysis 1: Excluding individuals with a follow-up duration of less than one year. (N=52,566)** | | | | | | |
| **<**29.83 | **0.96 (0.94, 0.99)** | **0.003** | **0.97 (0.95, 0.99)** | **0.006** | **0.97 (0.94, 0.99)** | **0.011** |
| $\boldsymbol{\geq}$29.83 | **1.03 (1.02, 1.04)** | **<0.001** | **1.03 (1.02, 1.04)** | **<0.001** | **1.04 (1.03, 1.05)** | **<0.001** |
| **Sensitivity analysis 2: Analysis based on the pre-imputation dataset (N=52,723)** | | | | | | |
| **<**29.83 | **0.96 (0.94, 0.99)** | **<0.001** | **0.97 (0.95, 0.99)** | **0.002** | **0.97 (0.94, 0.99)** | **0.004** |
| $\boldsymbol{\geq}$29.83 | **1.03 (1.02, 1.04)** | **<0.001** | **1.03 (1.02, 1.04)** | **<0.001** | **1.04 (1.03, 1.05)** | **<0.001** |
| **Sensitivity analysis 3： Adjusting for waist circumference on the basis of the original model 3. (N=52,723)** | | | | | | |
| **<**29.83 | **0.96 (0.94, 0.99)** | **<0.001** | **0.97 (0.95, 0.99)** | **0.002** | **0.97 (0.95, 0.99)** | **0.012** |
| $\boldsymbol{\geq}$29.83 | **1.03 (1.02, 1.04)** | **<0.001** | **1.03 (1.02, 1.04)** | **<0.001** | **1.02 (1.01, 1.04)** | **0.001** |

Model 1 was unadjusted, and Model 2 was adjusted for Sex and Age. Model 3 added other covariates to Model 2, including Diabetes, Hypertension, and Dyslipidemia.

Abbreviations: HR, Hazard Ratio; CI, Confidence Interval; METS-IR, the Metabolic Score for Insulin Resistance.
